# Disease features, treatments, and burden of palmoplantar pustulosis in Korea: The EPPPIK study

**DOI:** 10.1111/1346-8138.17702

**Published:** 2025-04-01

**Authors:** Seong Jin Jo, Byung Soo Kim, Dong Hyun Kim, Jong Hoon Kim, Chun Wook Park, Sang Wook Son, Jiyoung Ahn, Ji Yeoun Lee, Ki‐Heon Jeong, Youngdoe Kim, Jihye An, Chul Jong Park, Sang Woong Youn

**Affiliations:** ^1^ Department of Dermatology Seoul National University College of Medicine Seoul Korea; ^2^ Department of Dermatology Pusan National University School of Medicine Busan Korea; ^3^ Department of Dermatology CHA University Bundang Medical Center Seongnam Korea; ^4^ Department of Dermatology Gangnam Severance Hospital Seoul Korea; ^5^ Department of Dermatology Hallym University Kangnam Sacred Heart Hospital Seoul Korea; ^6^ Department of Dermatology Korea University Ansan Hospital Ansan Korea; ^7^ Department of Dermatology National Medical Center Seoul Korea; ^8^ Department of Dermatology Chungbuk National University Hospital Cheongju Korea; ^9^ Department of Dermatology Kyung Hee University Hospital Seoul Korea; ^10^ Medical Affairs Janssen Korea Ltd. Seoul Korea; ^11^ Department of Dermatology, Bucheon St. Mary's Hospital, College of Medicine The Catholic University of Korea Seoul Korea; ^12^ Department of Dermatology Seoul National University Bundang Hospital and Seoul National University College of Medicine Seongnam Korea

**Keywords:** burden, disease features, palmoplantar pustulosis, quality of life, treatment satisfaction

## Abstract

Palmoplantar pustulosis (PPP) is a chronic skin disease characterized by erythema, pustules, and desquamation of the palms and soles. PPP epidemiological data are scarce. Understanding disease characteristic is essential for effective management and to reduce burden. The objective of the study was to describe disease characteristics of PPP in Korea, including demographics, disease burden, and current clinical practice. This was a cross‐sectional, multicenter, noninterventional study conducted among 20 sites in Korea. Patients (aged ≥19 years) with a confirmed PPP diagnosis were examined and interviewed, collecting clinical and patient‐reported outcomes in 1 day. A total of 379 patients with PPP (mean age, 51.4 years; 39.3% male) were enrolled. The mean age at diagnosis was 47.9 years, and the mean duration of PPP was 3.2 years. Mean±standard deviation of Palmoplantar Pustulosis Area and Severity Index (PPPASI) score was 10.3±9.0; 33.8% of patients scored ≥12. PPPASI score was significantly higher with younger age (*p* = 0.0001) and nail involvement (*p* = 0.0002). Mean Dermatology Life Quality Index (DLQI) score was 12.0, and mean EuroQoL 5‐Dimension Health Questionnaire (EQ‐5D) score was 62.4 ± 19. Patients with PPPASI ≥12 had significantly higher DLQI (14.1 ± 7.3 vs 10.9 ± 7.6; *p* < 0.0001) and lower EQ‐5D (57.9 ± 18.1 vs 64.7 ± 19.2; *p* = 0.002) scores. The most common treatments in patients with PPPASI <12 were topicals (50.6%), conventional therapies (34.7%), and retinoids (32.7%); the most common treatments with PPPASI ≥12 were conventional therapies (37.5%), topicals (34.4%), and retinoids (29.7%). Conventional therapy users were “more satisfied” than nonusers (49.6% vs 39.0%; *p* = 0.05), with a numerical difference observed for biologics (41.8% vs 63.1%; *p* = 0.07). PPP is a debilitating disease that significantly diminishes overall quality of life. Despite most patients receiving treatment, PPP burden persists. Developing treatment guidelines and identifying more targeted and effective treatments would help alleviate symptoms, reduce severity, and improve overall quality of life.

## 1 INTRODUCTION

Palmoplantar pustulosis (PPP) is a chronic inflammatory skin disorder characterized by erythema, vesicles, pustules, scales, and abnormal desquamation of the palms and soles.^1^, ^2^, ^3^ Patients with PPP may develop additional clinical features, including nonpustular cutaneous eruptions, nail pitting, onycholysis, subungual pustules and nail dystrophy, and psoriatic arthritis.^2^, ^4^, ^5^, ^6^ Common symptoms include pruritus, burning sensations, and pain leading to difficulty in performing daily activities involving the hands and feet.^7^ PPP is associated with several comorbidities, such as cardiometabolic diseases, inflammatory arthritis, thyroid‐associated disease, obesity, and dyslipidemia,^2^, ^6^, ^8^ and psychosomatic disorders, fear, and anxiety are also reported.^6^, ^9^ Quality of life (QOL) for patients with PPP can be significantly impaired due to symptoms and disease burden.^7^, ^10^

There is a lack of consensus on the cause and pathogenesis of PPP and, while several factors have been proposed, mechanisms underlying the development and progression of PPP remain unclear. Molecular overlaps exist between the different inflammatory palmoplantar diseases^11^; however, the development of vesicles at the acrosyringium may be a characteristic of PPP.^3^ Components of both the innate and adaptive immune systems, as well as genetic predisposition, have also been suggested as contributing factors.^2^ Environmental stressors such as smoking, stress, infections, and pharmacotherapy may also contribute to the chronic inflammation experienced in patients with PPP.^2^

Understanding the epidemiology and disease characteristics of PPP is essential for accurate diagnosis, effective treatment, patient education, and improvement in QOL. While several studies have examined the epidemiology or clinical characteristics of PPP in different countries,^12^, ^13^, ^14^, ^15^, ^16^, ^17^, ^18^, ^19^ comprehensive global data remain limited. Also, previous analyses using health insurance databases to calculate prevalence may have misclassified the disease attributable to the use of nonvalidated codes and nonspecialists assigning codes.^15^, ^16^, ^17^ In Korea, PPP epidemiological data to date have only come from single‐center studies.^18^, ^19^, ^20^, ^21^, ^22^ Generalizability of results to the wider Korean population therefore cannot be assumed.

This study aimed to evaluate disease and patient characteristics of adult patients with PPP in Korea, including demographics, risk factors, and disease‐specific burden. We analyzed medical records and patient‐ and physician‐completed assessments across a wider cross‐section of Korean patients, extending beyond insurance claims database studies. Additionally, the study aimed to gain insights into current clinical practices and satisfaction with the treatment modalities used for managing PPP.

## 2 METHODS

### 2.1 Study design and patients

In this multicenter, cross‐sectional, observational study among 20 hospitals in Korea (Table S1), patient recruitment occurred from February 1 to August 31, 2021. Inclusion criteria were age 19 years or older, a confirmed diagnosis of PPP determined by a dermatologist based on characteristic clinical features and/or histopathologic findings, and provision of informed consent allowing for data collection and source data verification in accordance with local requirements. Patients with clear clinical characteristics of PPP were diagnosed as such, while ambiguous cases were confirmed through biopsy. Patients who received any investigational drug or used invasive investigational medical devices within the preceding 6 months were not eligible.

### 2.2 Data collection

Primary data sources were the medical records for each patient as well as patient‐ and physician‐completed questions, scales, and assessments. Demographic data collected included age, sex, body mass index (BMI), age of menarche (if female), blood pressure, smoking/drinking, and medical histories. Disease‐related characteristics, including age at diagnosis, prior skin biopsy (yes/no), family history of PPP, nail involvement, Palmoplantar Pustulosis Area and Severity Index (PPPASI) score, Physician's Global Assessment (PGA) score, and current treatment modalities for PPP (topical, phototherapy, systemic corticosteroids, retinoids, and immunosuppressants including conventional therapy and biologics), were collected. A variety of assessment tools measured patient‐reported outcomes (PROs), including the Dermatology Life Quality Index (DLQI), EuroQoL 5‐Dimension Health Questionnaire (EQ‐5D), Itch Numerical Rating Scale (NRS), Pain NRS, Psoriatic Arthritis Screening and Evaluation (PASE), Hospital Anxiety and Depression Scale, Work Productivity and Activity Impairment, experience of halting work due to disease (leave of absence for more than a month or resignation), and the Medication Satisfaction Questionnaire (MSQ).

### 2.3 Ethics consideration

This study was conducted in accordance with the ethical principles that have their origin in the Declaration of Helsinki and that are consistent with Good Clinical Practices and applicable regulatory requirements. The study protocol and amendments were reviewed and approved by an institutional review board at each institution (Table S1). Patients or their legal representatives provided their written informed consent to participate in the study.

### 2.4 Statistical methods

Given that the study was observational by design, all analyses relied on descriptive statistics using the collected variables. Continuous variables are presented as mean with standard deviation (SD), and categorical variables are presented as frequency with percentage.

For exploratory purposes, a subgroup analysis was performed according to disease severity, comparing PPPASI <12 and ≥12 groups. A patient with a PPPASI score of ≥12 is classified as having moderate to severe PPP. Where appropriate, the following tests, without missing data imputation, were used: the Wilcoxon rank sum and *t* test for continuous variables, Fisher's exact test, and analyses for categorical variables based on Pearson's chi‐square test. In addition, linear regression analysis was conducted to elucidate factors affecting PPPASI. Variables for which the *p* value of the univariable linear regression analysis was <0.1, or those reported as relevant in previous studies, were included in the multivariable linear regression analysis. All statistical analyses were performed using two‐sided tests, and results with *p* values <0.05 were considered statistically significant. All analyses were performed using the statistical software package SAS version 9.4 (SAS Institute Inc.).

## 3 RESULTS

### 3.1 Demographics and clinical characteristics of patients

A total of 379 patients with PPP were enrolled, with a mean age of 51.4 ± 13.0 years. Of these, 149 (39.3%) were male, indicating a female predominance. Mean BMI was 24.4 ± 4.1 kg/m^2^; 138 (59.0%) patients were classified as normal weight, while 69 (29.5%) and 19 (8.1%) patients were classified as overweight and obese, respectively. Lifestyle behaviors revealed that 223 (58.8%) patients were current or ex‐smokers (154 [40.6%] and 69 [18.2%], respectively), with a mean of 15.9 ± 14.9 pack‐years; 233 (61.5%) patients were identified as either current or past drinkers (Table 1).

**TABLE 1:** Demographics and clinical characteristics.

Characteristics	Total (*N* = 379)	PPPASI < 12	PPPASI ≥ 12	*p* Value
		(*n* = 251)	(*n* = 128)	
Age, years	51.4±13.0	52.8±13.0	48.6±12.6	0.001^*^
Male, *n* (%)	149 (39.3)	92 (36.7)	57 (44.5)	0.14
Menarche age (*N* = 87), years	14.3±1.2	14.2±1.3	14.4±1.1	0.75
BMI (*N* = 234), kg/m^2^	24.4±4.0	24.6±3.8	24.1±4.5	0.31
Underweight (<18.5 kg/m^2^), *n* (%)	8 (3.4)	4 (2.4)	4 (6.1)	0.39
Normal (18.5–24.9 kg/m^2^), *n* (%)	138 (59.0)	100 (59.5)	38 (57.6)	
Overweight (25.0–29.9 kg/m^2^), *n* (%)	69 (29.5)	52 (31.0)	17 (25.8)	
Obesity (≥30.0 kg/m^2^), *n* (%)	19 (8.1)	12 (7.1)	7 (10.6)	
SBP (*N* = 236), mm Hg	127.7±14.8	128.2±15.7	126.5±12.8	0.46
≥140 mm Hg, *n* (%)	35 (14.8)	23 (14.8)	12 (14.8)	0.10
Smoking status				
Current smoker, *n* (%)	154 (40.6)	99 (39.4)	55 (43.0)	0.62
Past smoker, *n* (%)	69 (18.2)	49 (19.5)	20 (15.6)	
Nonsmoker, *n* (%)	156 (41.2)	103 (41.0)	53 (41.4)	
Smoking pack‐year (*N* = 192)	15.9±14.9	15.9±14.0	15.9±16.6	0.47
Current/past drinking, *n* (%)	233 (61.5)	154 (61.4)	79 (61.7)	0.95
Age at PPP diagnosis (*N* = 351), years	47.9±13.3	49.1±13.0	45.5±13.5	0.009^*^
Duration of PPP (*N* = 351), years	3.2±4.3	3.4±4.5	2.8±4.0	0.05
Biopsy confirmed, yes, *n* (%)	161 (42.5)	120 (47.8)	41 (32.0)	0.003^*^
Family history of PPP, yes, *n* (%)	82 (21.6)	44 (17.5)	38 (29.7)	0.007^*^
Nail involvement, yes,^a^ *n* (%)	185 (48.8)	112 (44.6)	73 (57.0)	0.02^*^
Nail matrix	116 (30.6)	68 (27.1)	48 (37.5)	0.04^*^
Nail bed	140 (36.9)	79 (31.5)	61 (47.7)	0.002^*^
Concurrent with psoriasiform dermatitis, *n* (%)	62 (16.4)	36 (14.3)	26 (20.3)	0.14
Duration of psoriasiform dermatitis (*N* = 62), years	6.8±11.0	8.1±12.4	4.7±7.8	0.49
PPPASI	10.3±9.0	4.9±3.2	20.7±7.4	<0.0001^*^
PGA, *n* (%)				
0/1 (clear or almost clear)	80 (21.1)	74 (29.5)	6 (4.7)	<0.0001^*^
3/4/5 (moderate to very severe)	157 (41.4)	62 (24.7)	95 (74.2)	<0.0001^*^
Any comorbidities, *n* (%)	156 (41.2)	110 (43.8)	46 (35.9)	0.14
Hypertension, *n* (%)	57 (15.0)	40 (15.9)	17 (13.3)	0.49
Dyslipidemia,^b^ *n* (%)	45 (11.9)	38 (15.1)	7 (5.5)	0.006
Type 2 diabetes, *n* (%)	26 (6.9)	17 (6.8)	9 (7.0)	0.93
Any arthritis,^c^ *n* (%)	20 (5.3)	11 (4.4)	9 (7.0)	0.28
Anterior chest wall pain, *n* (%)	16 (4.2)	11 (4.4)	5 (3.9)	0.83
Psoriatic arthropathy, *n* (%)	8 (2.1)	4 (1.6)	4 (3.1)	0.45
Inflammatory bowel disease,^d^ *n* (%)	2 (0.5)	1 (0.4)	1 (0.8)	1.0
SAPHO syndrome, *n* (%)	3 (0.8)	1 (0.4)	2 (1.6)	0.27

*Note*: Values are presented as mean±standard deviation or number (percentage).

Abbreviations: BMI, body mass index; PGA, Physician's Global Assessment; PPP, palmoplantar pustulosis; PPPASI, Palmoplantar Pustulosis Area and Severity Index; SAPHO, synovitis/acne/pustulosis/hyperostosis/osteitis; SBP, systolic blood pressure.

^*^ Statistically significant result at a significance level of 0.05 between PPPASI <12 and PPPASI ≥12.

^a^ Any lesion in the nail matrix or nail bed.

^b^ Including dyslipidemia, hyperlipidemia, hypercholesterolemia, and hypertriglyceridemia.

^c^ Including psoriatic arthropathy, rheumatoid arthritis, ankylosing spondylitis, osteoarthritis, articular calcification, periarthritis, joint dislocation, arthralgia, spinal osteoarthritis, facet joint syndrome, and spinal stenosis.

^d^ Including ulcerative colitis and Crohn's disease.

Mean age at established diagnosis of PPP was 47.9 ± 13.3 years, with a mean disease duration of 3.2 ± 4.3 years; 161 (42.5%) patients had PPP confirmed by biopsy. Family history was reported by 82 (21.6%) patients. Nail manifestations were observed in 185 (48.8%) patients; 116 (30.6%) and 140 (36.9%) had nail matrix or nail bed involvement, respectively. Concurrent psoriasiform dermatitis was present in 62 (16.4%) patients. At the time of enrollment, overall mean PPPASI was 10.3 ± 9.0; 80 (21.1%) patients were graded as PGA 0/1 (clear or almost clear), whereas 157 (41.4%) were graded between PGA 3 and 5 (moderate to very severe) (Table 1).

Furthermore, beyond skin involvement, 156 (41.2%) patients reported having any comorbidity, with hypertension (57 patients [15.0%]) and dyslipidemia (45 patients [11.9%]) representing the most prevalent. A small proportion had psoriatic arthropathy (8 [2.1%]), and SAPHO syndrome (synovitis, acne, pustulosis, hyperostosis, osteitis) was diagnosed in three patients (0.8%; Table 1).

### 3.2 Comparison between mild and moderate to severe PPP

At enrollment, 251 (66.2%) patients had PPPASI <12, and 128 (33.8%) patients had PPPASI ≥12. Patients' mean age was significantly lower in the PPPASI ≥12 group (48.6 ± 12.6 vs 52.8 ± 13.0 years; *p* = 0.001), and mean age at onset was also lower in the PPPASI ≥12 group (45.5 ± 13.5 vs 49.1 ± 13.0; *p* = 0.009). Family history of PPP was more prevalent for patients in the PPPASI ≥12 group. Nail involvement, especially in the nail matrix, was also more pronounced in the PPPASI ≥12 group. However, differences in BMI categories, smoking status, alcohol consumption, disease duration, concurrent psoriasiform dermatitis, and prevalence of comorbidities were not statistically significant between the two groups (Table 1).

To elucidate risk factors affecting severity of PPP, a PPPASI regression model incorporating pivotal demographic and clinical factors identified as relevant in previous studies was established. Multivariable linear regression analysis revealed that a PPPASI score of ≥12 was significantly associated with younger age (*β* = −0.1; *p* < 0.001) and presence of nail involvement (*β* = 3.5; *p* < 0.001) (Table 2).

**TABLE 2:** Risk factors associated with PPPASI (linear regression).

Characteristics	Reference	Univariable			Multivariable		
		Beta	SE	*p* Value	Beta	SE	*p* Value
Age (years)	–	–	–	–	−0.1	0.03	<0.0001
Female	Male	–	–	–	−1.5	1.0	0.14
Smoking (past/current)	None	0.7	1.0	0.5	0.5	1.0	0.61
Family history	No	1.5	1.1	0.2	2.0	1.1	0.07
Nail involvement	No	3.5	0.9	0.0001	3.5	1.0	0.0002
With psoriasiform dermatitis	Without	2.4	1.2	0.05	2.3	1.2	0.06

*Note*: Variables with *p* value <0.1 in univariable regression, or those reported as relevant in the previous studies, were used for multiple regression. Age and sex were used for basic covariates in univariable and multivariable regression.

Abbreviations: PPPASI, Palmoplantar Pustulosis Area and Severity Index; SE, standard error.

### 3.3 Physical and psychological burden in patients with PPP

PROs demonstrated substantial impairment of QOL for patients with PPP; the mean DLQI score (*n* = 366) was 12.0 ± 7.6, with 193 (52.7%) patients having a score of ≥11, and the mean EQ‐5D overall health condition score was 62.4 ± 19.1. Patients with PPPASI ≥12 had significantly higher DLQI scores (14.1 ± 7.3 vs 10.9 ± 7.6; *p* < 0.0001) and lower EQ‐5D scores (57.9 ± 18.1 vs 64.7 ± 19.2; *p* = 0.002), indicating a greater impairment in QOL. Similarly, the proportion of patients reporting itch, pain, and psychological burden such as anxiety, and the percentages of patients reporting overall work and social activity impairment, were significantly higher in the PPPASI ≥12 group than the PPPASI <12 group. However, differences in depression score, proportion of suspected patients with psoriatic arthritis and PASE ≥37, and proportion of patients having a leave of absence due to PPP were not statistically significant between the two groups (Table 3).

**TABLE 3:** PROs and socioeconomic characteristics.

Category^a^	Total	PPPASI < 12	PPPASI ≥ 12	*p* Value
	(*N* = 379)	(*n* = 251)	(*n* = 128)	
DLQI (*N* = 366), score^b^	12.0±7.6	10.9±7.6	14.1±7.3	<0.0001*
DLQI ≥11, *n* (%)	193 (52.7)	114 (47.1)	79 (63.7)	0.003*
DLQI 0/1, *n* (%)	22 (6.0)	22 (9.1)	0 (0.0)	0.0001*
EQ‐5D overall health condition, score^c^	62.4±19.1	64.7±19.2	57.9±18.1	0.002*
Itching NRS, score^d^	3.8±3.0	3.4±3.0	4.6±2.9	0.0001*
Pain NRS, score^e^	3.3±3.1	2.9±3.0	4.2±3.1	<0.0001*
PASE (*N* = 375), score^f^	35.3±15.1	34.1±14.7	37.8±15.7	0.03*
PASE ≥37, *n* (%)	175 (46.7)	110 (43.8)	65 (52.4)	0.12
HADS Anxiety, score^g^	7.3±4.5	6.8±4.4	8.4±4.6	0.002*
HADS Depression, score^g^	7.9±4.2	7.8±4.2	8.2±4.2	0.39
WPAI overall work impairment (*N* = 164), %^h^	37.8±29.1	31.4±26.6	50.0±29.9	0.0002*
WPAI social activity impairment (*N* = 352), %^i^	46.2±31.0	42.1±31.6	54.7±27.9	0.0003*
Leave of absence due to disease (*N* = 362), *n* (%)	49 (13.5)	33 (13.6)	16 (13.3)	0.54

*Note*: Values are presented as mean±standard deviation or number (percentage).

Abbreviations: DLQI, Dermatology Life Quality Index; EQ‐5D, EuroQoL 5‐Dimension Health Questionnaire; HADS, Hospital Anxiety and Depression Scale; NRS, Numerical Rating Scale; PASE, Psoriatic Arthritis Screening and Evaluation; PPPASI, Palmoplantar Pustulosis Area and Severity Index; PRO, patient‐reported outcome; WPAI, Work Productivity and Activity Impairment.

^a^ Patients who had missing visit numbers were excluded.

^b^ DLQI is a 10‐question validated questionnaire that measures the degree to which a patient's life is affected by the skin condition, and the scores range from 0 to 30, with higher scores indicating greater impairment in quality of life.

^c^ The EQ‐5D descriptive system is a preference‐based health‐related quality‐of‐life measure with one question for each of the five dimensions that include mobility, self‐care, usual activities, pain/discomfort, and anxiety/depression. A visual analog scale is used to measure self‐perceived health state on a scale of 0 to 100, with 100 being the best imaginable state.

^d^ Itching NRS is a patient‐reported measure of itch severity in the past 24 h, with scores ranging from no (0 points), mild (1–3 points), moderate (4–6 points), severe (7–8 points), to very severe (≥9 points).

^e^ Pain NRS assesses pain intensity on a 0 to 10 ranking scale, with 0 representing no pain and 10 representing the most intense pain imaginable.

^f^ PASE is a 15‐item self‐administered questionnaire used to assess the risk for inflammatory arthritis with a total score of 75, and patients with higher scores are more likely to have psoriatic arthritis.

^g^ HADS is a 14‐item scale with seven items each for anxiety and depression subscales, with a subscale score of >8 indicating anxiety or depression.

^h^ WPAI overall work impairment measures the percentage of work time missed and the percentage of impairment experienced while at work due to health conditions.

^i^ WPAI social activity impairment measures the percentage of impairment in daily activities due to health conditions.

* Statistically significant result at a significance level of 0.05 between PPPASI <12 and PPPASI ≥12.

### 3.4 Current treatment modalities and patient satisfaction

The most employed treatment modalities were topical agents (171 [45.1%]), which included corticosteroids (87 [23.0%]), vitamin D (4 [1.1%]), and a fixed‐dose combination of a corticosteroid and vitamin D (98 [25.9%]). Conventional therapies were being used by 134 (35.4%) patients, including cyclosporine (113 [29.8%]) and methotrexate (22 [5.8%]). A total of 120 patients (31.7%) were currently using retinoids. Among these retinoid users, acitretin was the most common choice, with 93 patients (24.5%) using it. Additionally, biologics were being used by 19 patients (5.0%).

Significantly fewer patients were using topical agents in the PPPASI ≥12 group than in the PPPASI <12 group (44 [34.4%] vs 127 [50.6%]; *p* = 0.003), whereas phototherapy (18 [14.1%] vs 6 [2.4%]; *p* < 0.0001) and biologics (11 [8.6%] vs 8 [3.2%]; *p* = 0.02) were used more frequently in the PPPASI ≥12 group compared with the PPPASI <12 group. Retinoids were prescribed in approximately 30% of patients in both groups (Table 4).

**TABLE 4:** Current PPP treatments.

Treatment category, *n* (%)	Total (*N* = 379)	PPPASI < 12	PPPASI ≥ 12	*p* Value
		(*n* = 251)	(*n* = 128)	
Topical use	171 (45.1)	127 (50.6)	44 (34.4)	0.003*
Topical corticosteroid	87 (23.0)	68 (27.1)	19 (14.8)	0.01*
Topical vitamin D	4 (1.1)	3 (1.2)	1 (0.8)	1.00
Fixed‐dose combination of corticosteroid and vitamin D	98 (25.9)	68 (27.1)	30 (23.4)	0.44
Phototherapy	24 (6.3)	6 (2.4)	18 (14.1)	<0.0001*
Systemic corticosteroids	16 (4.2)	9 (3.6)	7 (5.5)	0.39
Retinoid	120 (31.7)	82 (32.7)	38 (29.7)	0.56
Acitretin	93 (24.5)	63 (25.1)	30 (23.4)	0.72
Others	27 (7.1)	19 (7.6)	8 (6.3)	0.64
Conventional therapy	134 (35.4)	87 (34.7)	47 (35.7)	0.69
Cyclosporine	113 (29.8)	72 (28.7)	41 (32.0)	0.50
Methotrexate	22 (5.8)	15 (6.0)	7 (5.5)	0.84
Biologics^a^	19 (5.0)	8 (3.2)	11 (8.6)	0.02*

Abbreviations: PPP, palmoplantar pustulosis; PPPASI, Palmoplantar Pustulosis Area and Severity Index.

^a^ Guselkumab (14), ixekizumab (2), adalimumab (1), secukinumab (1), ustekinumab (1).

* Statistically significant result at a significance level of 0.05 between PPPASI <12 and PPASI ≥12.

Of the 379 patients, 342 (90.2%) completed the MSQ. Responses indicating “somewhat satisfied,” “very satisfied,” or “extremely satisfied” were interpreted as expressions of satisfaction with treatment. Overall, the MSQ result was 43.0%. Between the PPPASI ≥12 and <12 groups, a comparable proportion of patients was satisfied with their current treatment (42.7% vs 43.2%; *p* = 0.95).

When evaluating satisfaction with a particular treatment, a significantly greater proportion of conventional therapy users was satisfied with their treatment than nonusers (83/213 [39.0%] vs 64/129 [49.6%]; *p* = 0.05). Patients using biologics numerically reported higher treatment satisfaction compared with those not using biologics (41.8% vs 63.1%). However, the difference between the two groups did not reach statistical significance (*p* = 0.07). The proportion of acitretin users who reported being satisfied with their treatment was numerically less compared with nonusers, but this difference did not reach statistical significance at the *p* = 0.05 level (31/90 [34.4%] vs 116/252 [46.0%]; *p* = 0.06); the trend was not seen for other retinoids (Table 5).

**TABLE 5:** Medication satisfaction^a^ by current treatment.

Treatment category, *n* (%)	Satisfied with medication (*N* = 342)		*p* Value
	Nonuser	User	
Topical use	74/172 (43.0)	73/170 (42.9)	0.99
Topical corticosteroid	115/255 (45.1)	32/87 (36.8)	0.18
Topical vitamin D	146/338 (43.2)	1/4 (25.0)	0.64
Fixed‐dose combination of corticosteroid and vitamin D	104/245 (42.4)	43/97 (44.3)	0.75
Phototherapy	139/320 (43.4)	8/22 (36.4)	0.52
Systemic corticosteroids for use	144/329 (43.8)	3/13 (23.1)	0.14
Retinoid	106/229 (46.3)	41/113 (36.3)	0.08
Acitretin	116/252 (46.0)	31/90 (34.4)	0.06
Others	137/319 (42.9)	10/23 (43.5)	0.96
Conventional therapy	83/213 (39.0)	64/129 (49.6)	0.05*
Cyclosporine	94/233 (40.3)	53/109 (48.6)	0.15
Methotrexate	135/321 (42.1)	12/21 (57.1)	0.18
Biologics^b^	135/323 (41.8)	12/19 (63.2)	0.07

Abbreviation: MSQ, Medication Satisfaction Questionnaire.

^a^ Medication satisfaction defined as “somewhat satisfied,” “very satisfied,” or “extremely satisfied” responses on the MSQ.

^b^ Guselkumab (14), ixekizumab (2), adalimumab (1), secukinumab (1), ustekinumab (1).

* Statistically significant result at a significance level of 0.05 between noncurrent user and current user.

## 4 DISCUSSION

Global PPP prevalence data are limited and vary among countries and regions. The prevalence of PPP has been reported to range between 0.005% and 0.09% in Europe^12^, ^13^, ^17^, ^23^ and from 0.009%^12^ to 0.03% in the United States,^14^ while the reported prevalence in Canada is much lower at 0.00015%.^24^ In contrast, the prevalence of PPP in Japan, 0.1%, was much higher,^15^, ^23^ and in Korea, PPP prevalence rates of 0.05% and 0.06% have been reported.^8^, ^16^, ^23^ Reasons for the varied prevalence are not clear, but could be attributable to differences in genetic backgrounds, race, or even study methodology.

In the present study, 60.7% of patients with PPP were female. This aligns with previous studies that reported a predominance of females among patients with PPP worldwide (ranging from 58% to 94%),^25^ and also with a study in Korea (65.2%).^21^ However, some Korean studies have reported a more equal sex distribution, ranging from 42.7% to 52.4%;^8^, ^18^, ^20^, ^22^ these studies had the limitations of being small in scale and only representing single sites. Racial differences, or culturally related environmental risk factors such as smoking status, may account for the high variability reported worldwide.

PPP is reported predominantly in middle‐aged patients. The age range of patients included in the present study is similar to what has been reported in the majority of relevant literature (age range, 20–69 years)^15^, ^25^, ^26^, ^27^ and other Korean studies.^18^, ^19^, ^20^, ^21^, ^22^ Notably, younger patients reported more severe PPP. Likewise, a younger age at onset has been previously reported to be associated with more severe PPP.^28^ Concurrent psoriasiform dermatitis was present in approximately one‐quarter of patients in our study, and the most commonly reported comorbidities, such as hypertension, dyslipidemia, and type 2 diabetes, were generally in line with what was seen in previous PPP studies.^6^, ^8^ It should be noted that the investigators reported psoriasiform dermatitis based on the observation of erythematous patches with well‐defined borders and scales. However, this concurrent skin condition was not histologically confirmed through biopsy, which should be further evaluated.

PPP is a challenging disease to diagnose as it shares many similar clinical and histological features with other inflammatory skin diseases, such as dyshidrotic eczema. Clear characterization of PPP histopathologic features (e.g. vesicles lacking spongiosis, microabscesses on the edge of vesicles, loss of granular layer) has been reported in studies by Masuda‐Kuroki et al. and Yoon et al., further improving the differential diagnosis of PPP.^20^, ^29^ However, there are currently no established standards of care or guidelines.^25^ Commonly used treatments include topical and systemic therapies, phototherapy, biologics, and other oral therapies.^25^, ^30^, ^31^ There is a lack of well‐documented clinical trial data to support a standardized treatment paradigm for PPP.^30^, ^32^ However, topical corticosteroids are often suggested for first‐line therapy or mild disease.^6^, ^31^ For patients with moderate to severe PPP, phototherapy or a systemic agent, such as a systemic retinoid, methotrexate, or cyclosporine, may be effective,^6^, ^31^ although variable and unpredictable clinical responses have been reported.^33^ Since many patients become refractory to or have contraindications with topical or traditional systemic medications, biologic therapies, such as tumor necrosis factor α, interleukin (IL) 17, and IL‐23 inhibitors, can be considered. Although there are no specific treatment guidelines for PPP in Korea, in general, topical treatments are commonly used for patients with mild PPP and systemic treatments are typically reserved for patients with moderate to severe PPP. Based on the results of a phase 3 trial, the IL‐23 inhibitor guselkumab was approved to treat PPP in Japan.^27^ In Korea, guselkumab is also approved for the treatment of PPP and is the only biologic currently approved in this setting.^34^, ^35^ In practice, guselkumab is reserved for adults with moderate or severe disease who have inadequate response to conventional treatments.

In this study, primarily topical treatments, conventional therapy (cyclosporine and methotrexate), and retinoids (predominantly acitretin) were used to treat PPP, reflecting what is generally used in clinical practice. Topical medications were used more frequently in patients with PPPASI <12, while biologics and phototherapy were used more commonly in those with PPPASI ≥12. Although the current study was cross‐sectional in design and did not directly evaluate the effectiveness of each treatment method, an indirect measure of treatment effectiveness may be inferred from patient satisfaction. Notably, the rate of patient satisfaction was found to be significantly higher in users of conventional therapies overall compared with nonusers. Despite the difference, it should be noted that the satisfaction rate was <50% among conventional therapy users, which indicates a need for improved treatment. Although patient satisfaction did not show a significant difference between users and nonusers of biologics, possibly due to the low proportion of biologics users (5.0%), 63.2% of biologic users were satisfied with treatment, which suggests that biologics may be a beneficial treatment option for some patients. Retinoids have held a longstanding recommendation for the treatment of PPP. However, the present study revealed relatively low levels of satisfaction with retinoid treatment, indicating that drugs in this class may be either less effective than previously assumed or their side effects may have a more substantial negative impact on patients' QOL and well‐being than previously recognized by healthcare professionals.

The overall disease burden of PPP is associated with a significant impairment of patient QOL due to symptoms and signs including pain, itchiness, burning, and bleeding fissures.^2^, ^7^, ^10^ The significant positive correlation found between PPPASI and DLQI further supports that severity of disease proportionately affects QOL in patients with PPP.^36^ Consistent with previous reports, this study found that PPP has a significant impact on QOL, with patients reporting high levels of symptoms and signs, including itch and anxiety, and a negative impact on work and social activity. These findings are representative of the burden and poor QOL experienced by patients with more severe PPP. In turn, clinicians should actively monitor the QOL of patients with PPP and recognize the negative impact of the disease on their overall well‐being. By regularly assessing PROs and considering the physical and psychosocial effects of PPP, healthcare providers can make better‐informed treatment decisions and optimize patient care.

The findings of this study have implications for the management of PPP. Understanding which individuals may be at higher risk of developing or progressing to more severe PPP would help clinicians prioritize care and provide appropriate interventions. This study found, for example, that more severe PPP may occur in younger patients, patients with nail involvement, and possibly in those with psoriasiform dermatitis. Considering these results, treatment options could be better optimized based on individual patients' disease profile, with systemic medications reserved for patients with, or who are at risk of, moderate to severe PPP. Milder cases may benefit from treatments with a better risk–benefit profile, such as the topical therapies.

There are limitations to consider when interpreting findings from this study, including its cross‐sectional design and absence of a control group. The relatively small sample size and focus on only the Korean population may impact the generalizability of the findings; however, it is worth noting that studies involving larger populations of patients with PPP are uncommon due to the rare nature of the disease. Last, reliance on self‐reported data, limited information on treatment outcomes, and the absence of a formal assessment of treatment responses should also be considered. Prospective follow‐up of patients to better understand the natural progression of PPP and treatment approaches used would be beneficial, especially considering the challenges associated with effectively managing this condition.

Studies related to the prevalence of PPP are lacking worldwide. This multicenter study in Korea provides comprehensive data on the demographic and disease characteristics of patients with PPP. PPP is a debilitating disease that has a significant impact on patients' daily activities and diminishes their overall QOL. Despite most patients receiving some form of treatment, the burden of PPP tends to persist for many. Development of more targeted and effective treatment options specifically designed for moderate to severe PPP is needed to more effectively alleviate symptoms, reduce disease severity, and improve overall QOL for patients. By recognizing the burden of disease and addressing it through better therapeutic interventions and support, healthcare providers can work towards enhancing the overall well‐being of patients with PPP.

## FUNDING INFORMATION

This study was funded by Janssen Korea Ltd. (study no. CNTO1959PPP4001).

## CONFLICT OF INTEREST STATEMENT

Seong Jin Jo received a grant from Janssen Pharmaceuticals for this study and reported receiving grants and/or personal fees for lectures or consultation outside of this study from AbbVie, Boehringer Ingelheim, Bristol Myers Squibb, Celltrion Healthcare, Daewoong Pharmaceutical, Eli Lilly and Company, Green Cross Laboratories, Janssen Pharmaceuticals, Kolon Pharma, LEO Pharma, Novartis, Pfizer, Sanofi, UCB, and Yuhan. Byung Soo Kim, Chul Jong Park, Chun Wook Park, Dong Hyun Kim, Ji Yeoun Lee, Jiyoung Ahn, Jong Hoon Kim, Ki‐Hoen Jeong, Sang Wook Son, and Sang Woong Youn received a grant from Janssen Pharmaceuticals for this study. Jihye An and Youngdoe Kim are full‐time employees of Janssen Korea Ltd.

## ETHICS STATEMENT

Approval of the research protocol and its amendments were obtained by an institutional review board at each institution (Table S1). Patients or their legal representatives provided their written informed consent to participate in the study. The study is registered as CNTO1959PPP4001.

## Supporting information


Data S1.

## Data Availability

The data sharing policy of Johnson & Johnson Innovative Medicine is available at https://www.janssen.com/clinical‐trials/transparency. As noted on this site, requests for access to the study data can be submitted through Yale Open Data Access (YODA) Project site at http://yoda.yale.edu.
