# Supplementary material for: Disease features, treatments, and burden of palmoplantar pustulosis in Korea: The EPPPIK study
Source: J Dermatol. 2025 Apr 1;52(5):802–11. doi: 10.1111/1346-8138.17702 (PMC12056274; doi:10.1111/1346-8138.17702)
Supplement: Supplementary file 1 — Data S1. [file JDE-52-802-s001.docx]

**Supplementary Material**

Supplemental eTable 1: Institution Name

| No. | Institution Name | IRB approval number |
| --- | --- | --- |
| 1 | Asan Medical Center | 2021-0348 |
| 2 | CHA Bundang Medical Center, CHA University | 2020-11-033 |
| 3 | Chosun University Hospital | 2021-01-008 |
| 4 | Chungbuk National University Hospital | 2021-01-023 |
| 5 | Chungnam National University Hospital | CNUH2020-12-076 |
| 6 | Gachon University Gil Medical Center | GDIRB2021-013 |
| 7 | Gangnam Severance Hospital | 2020-1004 |
| 8 | Hallym University Kangnam Sacred Heart Hospital | 2021-01-005 |
| 9 | Inje University Ilsan Paik Hospital | 2020-11-024 |
| 10 | Konkuk University Medical Center | KUMC2020-11-061 |
| 11 | Korea University Ansan Hospital | 2020AS0361 |
| 12 | Kyunghee University Medical Center | 2021-01-013 |
| 13 | National Medical Center | NMC-2021-05-069 |
| 14 | Pusan National University Hospital | 2101-011-099 |
| 15 | Samsung Medical Center | 2020-12-015 |
| 16 | Seoul National University Bundang Hospital | B-2012/657-305 |
| 17 | Seoul National University Hospital | H-2011-200-1177 |
| 18 | Severance Hospital | 2020-3667 |
| 19 | The Catholic University of Korea Bucheon St. Mary’s Hospital | HC20OSDI0120 |
| 20 | The Catholic University of Korea Seoul St. Mary's Hospital | KC21OSDE0076 |
